# Forward chemical genetic screens in Arabidopsis identify genes that influence sensitivity to the phytotoxic compound sulfamethoxazole

**DOI:** 10.1186/1471-2229-12-226

**Published:** 2012-11-24

**Authors:** Karl J Schreiber, Ryan S Austin, Yunchen Gong, Jianfeng Zhang, Pauline Fung, Pauline W Wang, David S Guttman, Darrell Desveaux

**Affiliations:** 1 Department of Cell & Systems Biology, University of Toronto, Toronto, ON, M5S 3B2, Canada; 2 Centre for the Analysis of Genome Evolution & Function, University of Toronto, Toronto, ON, M5S 3B2, Canada; 3 Current address: Department of Plant & Microbial Biology, University of California, Berkeley, CA, 94720-3102, USA; 4 Current address: Southern Crop Protection and Food Research Centre, Agriculture & Agri-Food Canada, London, ON, N5V 4T3, Canada

**Keywords:** Chemical genomics, Sulfanilamides, Arabidopsis thaliana

## Abstract

**Background:**

The sulfanilamide family comprises a clinically important group of antimicrobial compounds which also display bioactivity in plants. While there is evidence that sulfanilamides inhibit folate biosynthesis in both bacteria and plants, the complete network of plant responses to these compounds remains to be characterized. As such, we initiated two forward genetic screens in Arabidopsis in order to identify mutants that exhibit altered sensitivity to sulfanilamide compounds. These screens were based on the growth phenotype of seedlings germinated in the presence of the compound sulfamethoxazole (Smex).

**Results:**

We identified a mutant with reduced sensitivity to Smex, and subsequent mapping indicated that a gene encoding 5-oxoprolinase was responsible for this phenotype. A mutation causing enhanced sensitivity to Smex was mapped to a gene lacking any functional annotation.

**Conclusions:**

The genes identified through our forward genetic screens represent novel mediators of Arabidopsis responses to sulfanilamides and suggest that these responses extend beyond the perturbation of folate biosynthesis.

## Background

Sulfanilamide compounds occupy a prominent place in history as the first synthetic molecules to be employed as antimicrobial chemotherapeutics in clinical and veterinary practice [1]. Since their discovery in the 1930s, thousands of sulfanilamide derivatives have been synthesized and their mechanism of action studied extensively. Sulfanilamides are structural analogues of *p*-aminobenzoic acid (PABA) that competitively inhibit the enzyme dihydropteroate synthase (DHPS), which catalyzes a key step in the folate biosynthetic pathway [2]. Single base-pair changes in the DHPS gene can confer sulfanilamide resistance in bacteria [3], which has necessitated more selective utilization of these compounds in modern medicine.

Plants are also sensitive to sulfanilamides. In Arabidopsis, there is considerable evidence that these compounds inhibit the bifunctional folate biosynthetic enzyme hydroxymethyldiopterin pyrophosphokinase/dihydropteroate synthase (HPPK/DHPS) [4-7]. At low micromolar concentrations this inhibition is associated with reduced seedling growth, while higher concentrations are lethal. Overexpression of a sulfanilamide-insensitive bacterial DHPS in Arabidopsis renders these plants relatively insensitive to sulfanilamides [8]. This insensitivity is currently used as a selectable marker in some plant transformation vectors including those used to generate the GABI-Kat Arabidopsis T-DNA insertion collection [9]. Despite the characterization of sulfanilamide activity at the level of HPPK/DHPS, a broader understanding of plant responses to these compounds is lacking. Evidence of a more complicated response is suggested by the demonstration of folate-independent activities for a cytosolic isoform of HPPK/DHPS in Arabidopsis [7,10]. In addition, given that the folate biosynthetic pathway forms the foundation of one-carbon metabolism [11], sulfanilamide activity could affect a variety of important downstream metabolic components such as purines, amino acids, and enzyme cofactors. The influence of other metabolic pathways on sulfanilamide sensitivity is also unknown.

Here, we describe two forward genetic screens intended to dissect the responses of Arabidopsis to sulfanilamide compounds. We used seedling growth phenotypes to identify mutants with altered sensitivity to the compound sulfamethoxazole (Smex). A mutant with reduced sensitivity to Smex was mapped to the *OXOPROLINASE1* locus, while a mutant with enhanced sensitivity to Smex mapped to a gene of unknown function. Neither of these loci has previously been associated with sulfanilamide response phenotypes and thus represent novel mechanisms of altered sulfanilamide sensitivity.

## Results

### Seedling growth phenotypes and structure-activity analyses

**Figure 1:**
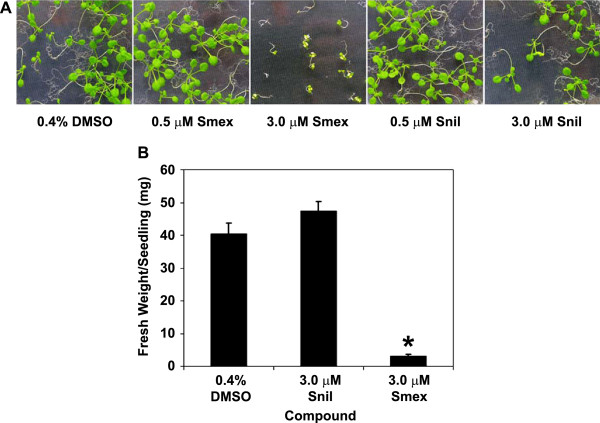
**Growth phenotypes of Arabidopsis seedlings germinated on media containing sulfamethoxazole (Smex).** (**a**) Seeds were germinated on Smex, sulfanilamide (Snil), or DMSO as a control. Images were captured after 16 days of growth. (**b**) Fresh weight measurements from seedlings grown in the presence of 0.4 % DMSO, 3 μM Smex, or 3 μM Snil. For each treatment, three plates were prepared with approximately 40 seeds each. Measurements were taken after 12 days of growth and represent the mean value from three plates ± standard deviation. The asterisk indicates a statistically significant difference between the Smex samples and the DMSO control as determined by a Student’s t-test (α=0.05). Similar results were obtained in at least two independent experiments.

**Figure 2:**
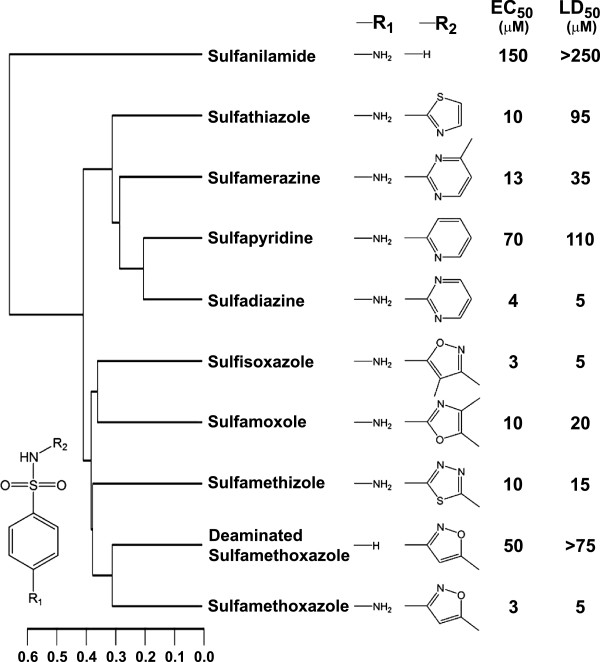
**Structure-activity relationships for various sulfanilamide compounds in a seedling growth assay.** The sulfanilamide core is shown in the bottom left corner, and specific R-groups for each compound indicated above. Activity assays were performed using Arabidopsis (Col-0) seed plated on solid media containing a range of chemical concentrations. Seedling phenotypes were assessed after 10–14 days of growth. “EC_50_” indicates the effective concentration of chemical at which 50% of the seedlings were bleached, and “LD_50_” denotes the concentration that was lethal to 50% of the seedlings. Data represent the mean of two independent experiments. The dendrogram on the left was generated using the ChemMine online structural analysis workbench ( http://bioweb.ucr.edu/ChemMineV2/).

In order to genetically dissect the activity of sulfanilamides in Arabidopsis, we required a phenotype amenable to high-throughput surveys. Such a phenotype was established based on the observation that Arabidopsis (Col-0) seeds germinated on solid media (0.5X MS, 2.5 mM MES; pH 5.8, 0.8% agar) containing 3 μM Smex yielded severely stunted seedlings that were almost completely bleached (Figure 1). We subsequently used the seedling growth phenotype to determine the structure-activity relationships amongst a larger set of sulfanilamides. When tested at 3 μM, the sulfanilamide core group itself did not significantly affect seedling growth (Figure 1). We also evaluated a number of additional sulfanilamide compounds (Figure 2) and noted a wide range of inhibitory activities in the seedling growth assay, with Smex being among the most active of all of the compounds tested. We observed that removal of the amino group from the sulfanilamide core (at position R_1_) severely compromised the activity of Smex in these assays. Interestingly, sulfanilamide itself exhibited even lower activity than deaminated Smex, suggesting that the major side group (R_2_) contributes significantly to chemical activity. It is clear, however, that there is considerable flexibility in the composition of this R-group with regards to effects on seedling growth. Both five- and six-member ring structures conferred similar activity, and this activity was generally retained despite a variety of substitutions within the ring and/or the presence of additional methyl groups at different positions around the ring. One major exception, however, was sulfapyridine which, despite differing from sulfadiazine by only one atom, exhibited drastically reduced activity. The chemical properties of these two ring structures are likely different, but their relation to activity remains unclear. We also noted a wide variation in the magnitude of differences observed between EC_50_ and LD_50_ values. In particular, four compounds had an EC_50_ of around 10 μM, yet the LD_50_ ranged from 15 to 95 μM. These observations may reflect different processes underlying the bleaching and lethality phenotypes used to determine EC_50_ and LD_50_ values, respectively. Additional phenotypic variation may derive from different rates of uptake of these chemicals by seedlings. Ultimately, docking models of each sulfanilamide with its target could assist in explaining this behavior.

### Mutants with reduced sensitivity to Smex

Based on the activity of Smex in the seedling growth assay, we initiated two forward chemical genetic screens, one of which involved germinating ethylmethanesulfonate-mutagenized Arabidopsis seeds on media containing 3 μM Smex in order to identify mutants with reduced sensitivity to Smex (RSS). From approximately 16,000 M_2_ seeds screened, 197 putative RSS mutants were identified, and reduced sensitivity to Smex was confirmed in either the M_3_ or M_4_ generation for 37 of these lines. In order to quantify the insensitivity of the mutants, each line was sown on media containing a range of Smex concentrations, revealing that nine mutants were significantly less sensitive to Smex than wildtype. Our criteria for judging significance stipulated that the concentrations of Smex required to inhibit seedling growth be at least 1.5 times higher than the concentration at which growth was inhibited in wildtype seedlings. The Arabidopsis genome contains two HPPK/DHPS genes [7], so the open reading frames of both genes were sequenced in all nine mutants, yet neither was found to contain mutations. Eight of the nine selected RSS lines were also less sensitive to the antifolate compound methotrexate. The lone exception, designated RSS 26–1, exhibited the greatest reduction in Smex sensitivity while remaining as sensitive as wildtype to methotrexate. This mutant also resembled wildtype plants in terms of its sensitivity to other phytotoxins such as salicylic acid and the herbicide Bialaphos (Table 1). 

**Table 1:** Phytotoxin sensitivity of wildtype Arabidopsis and a mutant that exhibits reduced sensitivity to sulfamethoxazole (RSS 26–1)

**Genotype**	**Compound**	**EC**_**50**_**(μM)**^**a**^	**LD**_**50**_**(μM)**^**b**^
Wildtype	Sulfamethoxazole	3	5
RSS 26-1	Sulfamethoxazole	6	10
Wildtype	Salicylic acid	75	150
RSS 26-1	Salicylic acid	75	150
Wildtype	Bialaphos	0.1	0.75
RSS 26-1	Bialaphos	0.1	0.75
Wildtype	Methotrexate	0.03	0.1
RSS 26-1	Methotrexate	0.03	0.1

^a^EC_50_ indicates the concentration at which 50% of seedlings are bleached.

^b^LD_50_ indicates the concentration that is lethal to 50% of seedlings.

Data represent observations from at least three independent experiments.

**Figure 3:**
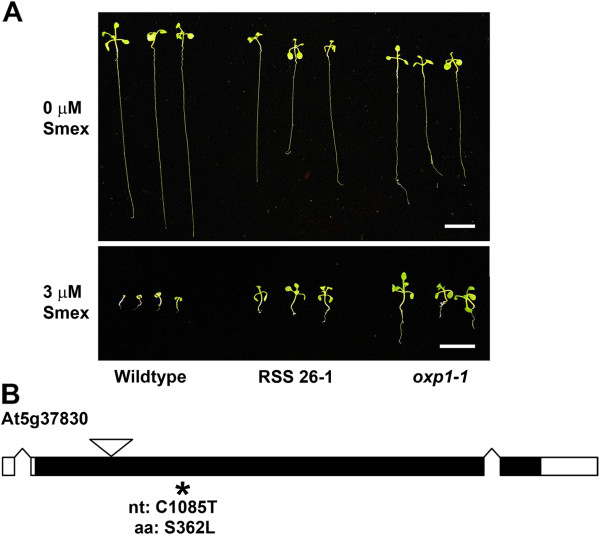
**Characterization of a “reduced sensitivity to sulfamethoxazole (Smex)” mutant (RSS 26–1).** (**a**) Seedling growth phenotypes of RSS 26–1 and an *oxoprolinase1* T-DNA insertion line (*oxp1-1*) on media containing 3 μM Smex. Images were captured after 14 days of growth. Scale bar represents 1 cm. (**b**) Gene model of At5g37830 (encoding OXOPROLINASE1). Black regions indicate open reading frames while white areas designate untranslated regions. The location of the point mutation mapped in this study for RSS 26–1 is indicated by an asterisk, and the specific sequence change is shown at both the nucleotide (nt) and amino acid (aa) levels. An inverted triangle denotes the approximate location of the T-DNA insertion (SALK_078745C, *oxp1-1*) used to confirm the RSS phenotype.

The apparent specificity of chemical insensitivity in RSS 26–1 suggested that the mutation underlying this phenotype should provide some insight into Smex-influenced pathways in Arabidopsis independent of folate metabolism. In preparation for mapping this mutation, RSS 26–1 (M_4_) was crossed to ecotype Landsberg *erecta* (L*er*). Smex-insensitive individuals from the F_2_ of this cross were recovered on media containing 5 μM Smex, and genomic DNA was extracted from the tissues of 80 individuals. Whole-genome sequencing was performed on an Illumina Genome Analyzer and the resulting data analyzed using a bioinformatics pipeline devised by Austin *et al*. [12] in order to identify a genomic region in which putative mutations of interest reside. Briefly, the frequency of Col-0/L*er* single nucleotide polymorphisms (SNPs) is evaluated across the genomic sequence, and the region of interest is defined by an enrichment of Col-0 SNPs representing the original mutant background. Within this region, mutations in or near coding sequences that are present in 100% of the sequences analyzed are considered mutations of interest. This analysis generated a short list of candidate genes whose relevance to the RSS phenotype was evaluated using T-DNA insertion lines (Table 2). These efforts confirmed that RSS 26–1 was derived from a mutation (C→T at nucleotide 1,085 of the coding sequence; S362L at the amino acid level) in a gene encoding an oxoprolinase enzyme (*OXP1*; At5g37830 [GenBank: NM_123142]). A homozygous *oxp1* knockout line (*oxp1-1*) resembled RSS 26–1 with regards to Smex sensitivity (Figure 3). 

**Table 2:** Characterization of candidate loci identified through mapping of a “reduced sensitivity to sulfamethoxazole” (RSS) phenotype

**Locus**	**Mutations Identified by Mapping**				**T-DNA Lines for Phenotypic Confirmation**	
	**Nucleotide Change**^**a**^	**Strand**	**Genome Position**^**b**^	**Amino Acid Change**	**Line(s) Examined**	**Phenotype on 3 μM Sulfamethoxazole**^**c**^
At5g37160	G → A	+	14705775	V90M	SALK_045992C, SALK_129697C	Bleached
At5g37830	C → T	-	15059441	S362L	SALK_078745C	Green
At5g39040	G → A	+	15629182	G529E	SALK_011884	Bleached
Wildtype	n/a	n/a	n/a	n/a	n/a	Bleached
RSS 26-1	n/a	n/a	n/a	n/a	n/a	Green

^a^Candidate loci were identified as described in *Methods*. The nucleotide change is shown in the context of the coding sequence, independent of strand orientation.

^b^Genome position as defined by the Arabidopsis Information Resource.

^c^Seedling phenotypes were evaluated after 14 days of growth on media containing 3 μM sulfamethoxazole. “Bleached”/”Green” describe the appearance of the majority of seedlings for each genotype and represent assessments from at least two independent experiments.

n/a = not applicable.

### Mutants with enhanced sensitivity to Smex

**Figure 4:**
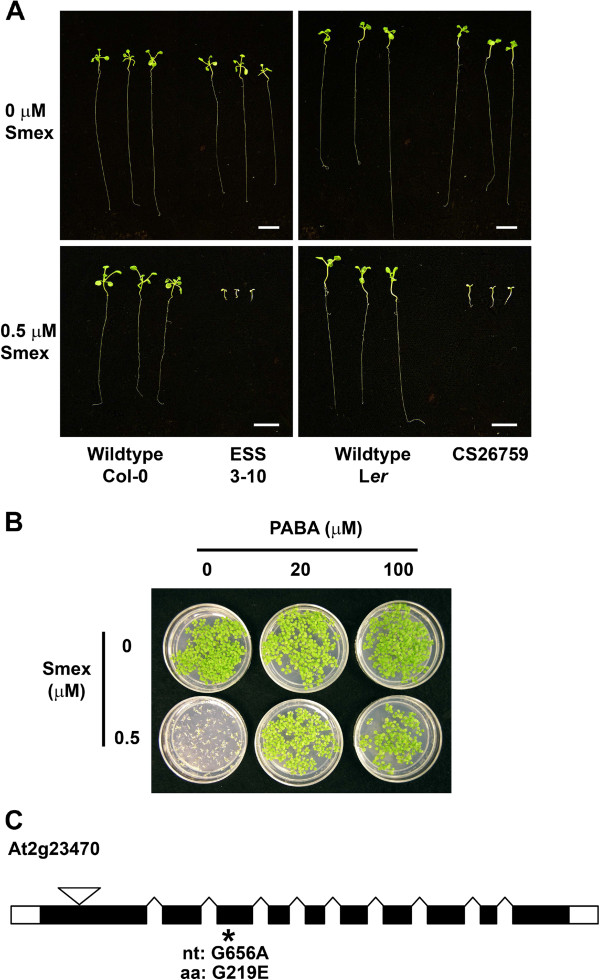
**Phenotypes associated with an “enhanced sensitivity to sulfamethoxazole (Smex)” mutant (ESS 3–10).** (**a**) Seedling growth phenotype of ESS 3–10 on media containing 0.5 μM Smex. The T-DNA insertion line CS26759 (in a Landsberg *erecta* background) is shown for comparison. Scale bar represents 1 cm. (**b**) Seedling growth phenotypes of ESS 3–10 on media containing both *p*-aminobenzoic acid (PABA) and 0.5 μM Smex. Both images were captured after 14 days of growth. (**c**) Gene model of At2g23470. Black regions indicate open reading frames while white areas designate untranslated regions. The location of the point mutation mapped in this study for ESS 3–10 is indicated by an asterisk, and the specific sequence change is shown at both the nucleotide (nt) and amino acid (aa) levels. An inverted triangle denotes the approximate location of the T-DNA insertion (stock CS26759) used to confirm the ESS phenotype.

In addition to screening for reduced sensitivity to Smex, a second forward genetic screen focused on identifying mutants with enhanced sensitivity to this compound. In this screen, seeds were germinated on media containing 0.5 μM Smex, which does not affect the growth or appearance of wildtype seedlings. Approximately 12,000 M_2_ seeds were screened, and 538 individuals exhibited bleaching and/or stunted growth. A secondary screen conducted on media containing either 0.5 μM Smex or 0.4% DMSO confirmed that three mutants displayed Smex-specific phenotypes. Complementation crosses indicated that all three mutants were recessive and allelic. We focused on “enhanced sensitivity to Smex” (ESS) mutant 3–10, which showed nearly 700-fold greater sensitivity to Smex than did wildtype Arabidopsis (Col-0) (Figure 4a). This phenotype could be partially rescued by the addition of PABA or dihydrofolate (DHF) (Figure 4b, data not shown), indicating that HPPK/DHPS inhibition contributes to Smex hypersensitivity in this mutant. Notably, the chemical hypersensitivity of ESS 3–10 was not a general phenomenon, because it did not differ from wildtype when germinated in the presence of salicylic acid, Bialaphos, or methotrexate (Table 3). As with RSS 26–1, a mapping population was generated for ESS 3–10 and sequenced *en masse*. Subsequent sequence analysis yielded a list of five candidate genes for which T-DNA knockout lines were obtained (Table 4). Seedling growth assays were then performed, revealing Smex hypersensitivity in a line bearing a T-DNA insertion within At2g23470 [GenBank: NM_127911]. Based on our sequencing data, this locus contained a G→A mutation at nucleotide 656 of the coding sequence, resulting in a G219E amino acid substitution in ESS 3–10.

**Table 3:** Phytotoxin sensitivity of wildtype Arabidopsis and a mutant that exhibits enhanced sensitivity to sulfamethoxazole (ESS 3–10)

**Genotype**	**Compound**	**EC**_**50**_**(μM)**^**a**^	**LD**_**50**_**(μM)**^**b**^
Wildtype	Sulfamethoxazole	3	5
ESS 3-10	Sulfamethoxazole	0.003	0.03
Wildtype	Sulfanilamide	150	>250
ESS 3-10	Sulfanilamide	5	30
Wildtype	Salicylic acid	75	150
ESS 3-10	Salicylic acid	75	150
Wildtype	Bialaphos	0.1	0.75
ESS 3-10	Bialaphos	0.1	0.75
Wildtype	Methotrexate	0.03	0.1
ESS 3-10	Methotrexate	0.03	0.1

^a^EC_50_ indicates the concentration at which 50% of seedlings are bleached.

^b^LD_50_ indicates the concentration that is lethal to 50% of seedlings.

Data represent observations from at least three independent experiments.

**Table 4:** Characterization of candidate loci identified through mapping of an “enhanced sensitivity to sulfamethoxazole” (ESS) phenotype

**Locus**	**Mutations Identified by Mapping**				**T-DNA Lines for Phenotypic Confirmation**	
	**Nucleotide Change**^**a**^	**Strand**	**Genome Position**^**b**^	**Amino Acid Change**	**Line(s) Examined**	**Phenotype on 0.5 μM Sulfamethoxazole**^**c**^
At2g23470	G → A	-	10000583	G219E	CS26759	Bleached
At2g24590	G → A	+	10450926	n/a^d^	SALK_023090, SALK_032699C, SALK_094266	Green
At2g25320	G → A	-	10785456	G732R	SALK_072877C, SALK_007242C	Green
At2g26135	C → T	+	11130249	A43V	SALK_150146C, SALK_055187	Green
At2g27790	C → T	-	11847919	T529I	SAIL_1155_B02, SAIL_1155_E08, SALK_062215C	Green
Wildtype	n/a	n/a	n/a	n/a	n/a	Green
ESS 3-10	n/a	n/a	n/a	n/a	n/a	Bleached

^a^Candidate loci were identified as described in *Methods*. The nucleotide change is shown in the context of the coding sequence, independent of strand orientation.

^b^Genome position as defined by the Arabidopsis Information Resource.

^c^Seedling phenotypes were evaluated after 14 days of growth on media containing 3 μM sulfamethoxazole. “Bleached”/”Green” describe the appearance of the majority of seedlings for each genotype and represent assessments from at least two independent experiments.

n/a = not applicable.

## Discussion

In addition to the interaction between a chemical and its direct target(s), a wide range of factors influence the activity of small molecules in biological systems [13]. The antimicrobial activity of the sulfanilamide family of compounds is attributed to the inhibition of bacterial DHPS. Although humans lack DHPS, sulfanilamides can bind to carbonic anhydrases and serum albumin, occasionally with toxic side effects [14-16]. In plants, folate-independent activities are also documented for HPPK/DHPS [7,10]. These observations, in combination with the broad array of metabolic pathways downstream of DHPS and folate biosynthesis, stimulated us to investigate the genes that influence the activity of sulfanilamides in plants.

To facilitate this investigation, we sought a readily scorable phenotype with which to assess the sensitivity of Arabidopsis seedlings to Smex. We found that germination on agar media supplemented with 3 μM Smex resulted in significant stunting and bleaching of wildtype (Col-0) seedlings. This phenotype was conducive to forward genetic screens focused on identifying Arabidopsis mutants with altered responses to Smex. A screen for mutants with reduced sensitivity to Smex yielded a number of hits that were significantly and quantitatively less responsive to Smex. Notably, while it is possible to confer insensitivity to sulfanilamides by a single base pair change in the DHPS gene [3,8], no mutations within the HPPK/DHPS sequences were observed with the mutants of interest. Also notable was the observation that most of these mutants were less sensitive to both Smex and methotrexate, another inhibitor of folate biosynthesis. Such a broad effect on plant responses to two different antifolate compounds may represent a compensatory mutation that increases overall flux through the folate biosynthetic pathway, reduces the uptake of chemicals from the surrounding media, or alters the activity of vacuolar ATP-binding cassette proteins, some of which are known to transport folates and methotrexate [17]. Only one mutant exhibited reduced sensitivity exclusively to Smex, and the relevant mutation was mapped to a gene encoding an oxoprolinase enzyme (*OXP1*).

In plants, oxoprolinase catalyzes the conversion of 5-oxoproline (pyroglutamic acid) into glutamate as part of a glutathione recycling pathway. Theoretically, the loss of OXP1 function could lead to an accumulation of glutathione consequent with an enhanced capacity for elimination of xenobiotics by conjugation to this molecule [18]. In reality, however, the overall composition and concentrations of thiols appear to be unaffected in *oxp1-1* mutants [19], so the RSS phenotype is likely not the result of increased Smex detoxification. This conclusion also agrees with our previous observations that Smex does not appear to be degraded or otherwise structurally modified *in planta* (Dr. Sean Cutler, personal communication). The loss of OXP1 is associated with other metabolic changes, including the accumulation of significant amounts of 5-oxoproline and reduced (14-30% lower) concentrations of glutamate in leaves [19]. There is some evidence that 5-oxoproline causes lipid and protein oxidation in animal neural tissues [20], but the extent to which this occurs in plants is unknown. The paucity of information on the physiological roles of 5-oxoproline in plants makes it difficult to hypothesize about the connection between *OXP1* and Smex activity in Arabidopsis.

A second screen focused on the identification of mutants with enhanced sensitivity to Smex. A highly hypersensitive mutant was recovered from this screen and mapped to a heretofore uncharacterized locus (At2g23470). This locus encodes a protein of unknown function whose only annotation relates to a sequence motif shared with RUS (root UV-B sensitive) proteins that control growth responses to UV light [21]. Interestingly, while Smex hypersensitivity could be rescued by co-treatment with the folate precursor PABA, the sensitivity of ESS 3–10 to methotrexate was not altered relative to wildtype. Consequently, it appears that Smex and ESS 3–10 influence both folate-dependent and -independent responses.

## Conclusions

We have described two forward genetic screens that were initiated to investigate plant responses to the sulfanilamide family of chemicals. We identified mutants with altered sensitivities to the compound Smex and discovered that a mutation at locus At5g37830 conferred reduced sensitivity to Smex, while a mutation within locus At2g23470 resulted in enhanced sensitivity. Importantly, this is the first demonstration of such phenotypes for either of the two genes. While the precise roles of At5g37830 and At2g23470 in mediating responses to Smex remain to be fully characterized, our preliminary data indicate that Smex can mediate multiple non-overlapping phenotypes in Arabidopsis.

## Methods

### Plant materials and bacterial strains

*Arabidopsis thaliana* (ecotype Columbia-0) was used for the seedling growth assays. Forward genetic screens were conducted with ethylmethanesulfonate-mutagenized Arabidopsis (Col-0) seeds (Lehle Seeds, Round Rock, TX, USA). For candidate gene analyses, the following loci and their associated T-DNA insertion lines were examined: At5g37160 (SALK_045992C, SALK_129697C), At5g39040 (SALK_011884), At5g37830 (SALK_078745C), At2g26135 (SALK_150146C, SALK_055187), At2g24590 (SALK_023090, SALK_032699C, SALK_094266), At2g25320 (SALK_072877C, SALK_007242C), At2g27790 (SAIL_1155_B02, SAIL_1155_E08, SALK_062215C), and At2g23470 (CS26759).All lines were obtained from the Arabidopsis Biological Resource Center (ABRC). Stock CS26759 is in a Landsberg *erecta* background.

Seeds for soil-grown plants were placed on moist soil (ProMix BX, Premier Horticulture Ltd., Dorval, PQ, Canada) amended with 20-20-20 fertilizer, stratified for four days at 4°C, then placed in a growth room with a nine-hour photoperiod and a day/night temperature regime of 22°C/18°C. For seedling growth assays, surface-sterilized seeds were plated on media composed of 0.5X Murashige and Skoog (MS) basal salts (SigmaAldrich, Oakville, ON, Canada), 2.5 mM 2-(N-Morpholino)ethanesulfonic acid (MES), pH 5.8 (SigmaAldrich), and 0.8% agar. Following four days of stratification at 4°C, plates were incubated at 22°C under continuous light.

### Genetic mapping by whole-genome sequencing

To map the mutations responsible for altered sensitivity to Smex, mutants of interest were crossed to ecotype Landsberg *erecta*. For each mutant, at least 100 F_2_ plants were selected on the basis of Smex sensitivity in seedling germination assays and transferred to soil. For ESS 3–10, stunted and bleached seedlings were rescued on 0.5X MS media containing 1.5% glucose prior to transplanting on soil. After three weeks of growth, tissues from 80 plants were pooled and genomic DNA was extracted using a Gentra Puregene Genomic DNA Extraction Kit (QIAGEN Inc., Mississauga, ON, Canada). Samples were sequenced on an Illumina Genome Analyzer IIx (Illumina, Inc., San Diego, CA, USA) according to the manufacturer’s protocol (Pauline Fung and Jianfeng Zhang, Centre for the Analysis of Genome Evolution and Function (CAGEF), University of Toronto, ON, Canada). Sequence assembly and analysis were also performed at CAGEF (Yunchen Gong and Dr. Ryan Austin). A list of candidate loci potentially associated with the Smex sensitivity phenotype was subsequently generated using the web-based application, Next-Gen Mapping ( http://bar.utoronto.ca/NGM; [12]). Raw Illumina reads from each sequenced mutant population were deposited in the Dryad Digital Repository (doi: http://10.5061/dryad.3sk8). Candidates were confirmed using homozygous T-DNA insertion lines germinated on 0.5X MS agar media containing the appropriate concentration of Smex.

## Abbreviations

DHF: Dihydrofolate; DHPS: Dihydropteroate synthase; ESS: Enhanced sensitivity to sulfamethoxazole; PABA: *p*-aminobenzoic acid; RSS: Reduced sensitivity to sulfamethoxazole; Smex: Sulfamethoxazole.

## Competing interests

The authors declare that they have no competing interests.

## Authors’ contributions

KJS performed the genetic screens, prepared the mapping populations, and carried out the sulfanilamide structure-activity analyses. JZ, PF, and PW were responsible for the whole-genome sequencing, while RA, YG, and DSG formatted and analyzed the sequencing output. DD conceived the project and, along with KJS, prepared the manuscript. All authors read and approved the final manuscript.
